# Effects of Colchicine Treatment on Bioactive Compounds and Structural Characteristics of the Epidermis and Cortex of Edible Cactus [*Opuntia ficus-indica* (L.) Mill.] Cladodes at Different Maturity Stages

**DOI:** 10.3390/foods15183310

**Published:** 2026-09-18

**Authors:** Kriangsuk Boontiang, Natthakitti Boonnak, Parinya Boonarsa, Sirithon Siriamornpun

**Affiliations:** 1 Department of Agricultural Technology, Faculty of Technology, Mahasarakham University, Kantarawichai, Maha Sarakham 44150, Thailand; kriangsuk.b@msu.ac.th (K.B.); 67010861001@msu.ac.th (N.B.); 2 Research Unit of Thai Food Innovation (TFI), Mahasarakham University, Kantarawichai, Maha Sarakham 44150, Thailand; 3 Department of Food Technology and Nutrition, Faculty of Technology, Mahasarakham University, Kantarawichai, Maha Sarakham 44150, Thailand; 68010862501@msu.ac.th

**Keywords:** colchicine, phenolic acids, flavonoids, chlorogenic acid, malic acid, functional foods

## Abstract

This study investigated the effects of colchicine treatment on phenolic and flavonoid profiles, organic acids, antioxidant activities, and structural characteristics of the epidermis and cortex of edible cactus [*Opuntia ficus-indica* (L.) Mill.] cladodes at different maturity stages (1, 3, and 6 months). Cladodes were treated with 0, 0.5, and 1.0% colchicine for 24 h prior to cultivation. Colchicine significantly influenced metabolite accumulation, with responses varying across tissues and maturity stages. In the epidermis, total phenolic content (TPC) ranged from 942.67 to 1292.72 mg GAE/100 g DW, while total flavonoid content (TFC) ranged from 509.42 to 1322.55 mg QE/100 g DW. HPLC analysis further revealed marked increases in chlorogenic acid (6.25-fold), gentisic acid (3.47-fold), and gallic acid (63.9%), while rutin and apigenin were the predominant flavonoids. In contrast, the cortex accumulated higher levels of organic acids, particularly malic acid. FTIR and XRD analyses indicated that colchicine treatment did not substantially alter the major functional groups or crystalline characteristics of the cladodes. Pearson’s correlation and heatmap analyses further revealed positive associations among phenolic compounds, flavonoids, and antioxidant activities. These findings indicate that colchicine concentration and maturity stage were associated with distinct patterns of metabolite accumulation and antioxidant activity in edible cactus cladodes, supporting their potential application as functional food ingredients.

## 1. Introduction

The increasing demand for sustainable and health-promoting food sources has intensified interest in resilient crops that can provide nutritional and functional benefits under challenging environmental conditions [1,2]. Among these crops, edible cactus has attracted increasing attention because of its adaptability to arid environments and its potential as a sustainable food resource.

Cactus (*Opuntia* spp.), a genus in the family Cactaceae, comprises more than 300 species, with *Opuntia ficus-indica* (L.) Mill. being one of the most economically important species [3,4]. This species exhibits strong adaptability to arid environments, making it a promising candidate for sustainable food production under changing climatic conditions [5]. Its edible cladodes contain approximately 80–95 g/100 g moisture, 1–2 g/100 g dietary fiber, and 0.5–1 g/100 g protein on a fresh-weight basis, while also providing minerals, vitamins, and diverse phytochemicals [6,7]. Importantly, the cladodes are recognized as a rich source of phenolic compounds and flavonoids. Reported total phenolic contents range from approximately 5–14 mg GAE/g dry weight (DW) in young cladodes to 0.3–12 mg GAE/g DW in older cladodes, while total flavonoid contents have been reported at approximately 6–13 mg CE/g DW depending on cultivar [8,9]. Detailed chromatographic profiling has further identified diverse phenolic constituents in *O. ficus-indica* cladodes, including flavonoids and phenolic acids, which are associated with antioxidant and potential health-promoting activities [9]. However, the accumulation of these compounds can vary with genetic background, environmental conditions, tissue type, and maturity stage [8]. Understanding these variations is therefore important for optimizing the functional value and utilization of edible cactus as a food ingredient.

Colchicine has been widely used as an antimitotic agent in plant research and breeding, and its application can induce physiological and biochemical changes in plants [10]. By interfering with spindle fiber formation during mitosis, colchicine can affect cell division and has been used to induce chromosome doubling and polyploidy in various plant species [11,12]. In cactus species, previous studies have demonstrated that colchicine treatment can induce morphological, anatomical, and cytological changes. In *Gymnocalycium mihanovichii*, colchicine treatment altered plant morphology and stomatal characteristics [13]. More recently, our study on *O. ficus-indica* confirmed the induction of tetraploid and cytochimeric plants following colchicine treatment and reported treatment-dependent changes in morphological traits, bioactive compounds, and antioxidant activities [14]. Beyond its effects on chromosome number, colchicine treatment has also been reported to influence plant growth, physiological responses, and the accumulation of secondary metabolites, including phenolic compounds, flavonoids, and antioxidant activities [15,16]. Such effects have been reported in *Stevia rebaudiana* Bertoni [16] and *Cyclocarya paliurus* (Batal.) Iljinskaja [15]. These findings indicate that colchicine treatment can influence both structural and biochemical characteristics of plants. However, the tissue-specific responses of *O. ficus-indica* cladodes to colchicine treatment across different maturity stages remain poorly understood. In particular, limited information is available on how colchicine concentration affects phenolic and flavonoid profiles, antioxidant activities, and organic acid accumulation in the epidermis and cortex during cladode maturation.

Based on these findings, we hypothesized that colchicine treatment would produce concentration- and maturity stage-dependent changes in phytochemical accumulation and antioxidant activities, with distinct metabolite profiles between the epidermis and cortex. Therefore, this study aimed to evaluate the effects of different colchicine concentrations on plant growth, bioactive compounds, antioxidant activities, metabolite profiles, and structural characteristics of *O. ficus-indica* cladodes at different maturity stages. These findings provide insights into the relationships among colchicine concentration, maturity stage, tissue type, and the functional quality of edible cactus cladodes, supporting their potential application as functional food ingredients.

## 2. Materials and Methods

### 2.1. Material and Chemicals

Cladodes of *O. ficus-indica* (L.) Mill. were collected from the MSU Cactus Greenhouse, Department of Agricultural Technology, Faculty of Technology, Mahasarakham University, Thailand, a facility registered under the Convention on International Trade in Endangered Species of Wild Fauna and Flora (CITES) (CITES No. P–TH–00428).

All reagents and standards used in this study were of analytical or HPLC grade and were purchased from Sigma-Aldrich Co. (St. Louis, MO, USA), unless otherwise stated. The reagents included Folin–Ciocalteu reagent, sodium carbonate (Na_2_CO_3_), sodium nitrite (NaNO_2_), aluminum chloride hexahydrate (AlCl_3_·6H_2_O), sodium hydroxide (NaOH), and 2,2–diphenyl–1–picrylhydrazyl (DPPH), together with the reagents required for the FRAP assay. HPLC-grade reference standards (≥90% purity) were used for the determination of phenolic acids, flavonoids, and organic acids. The phenolic acid standards included caffeic acid, chlorogenic acid, cinnamic acid, ferulic acid, gallic acid, gentisic acid, *p*-coumaric acid, *p*-hydroxybenzoic acid, protocatechuic acid, sinapic acid, syringic acid, and vanillic acid. The flavonoid standards included apigenin, kaempferol, myricetin, quercetin, and rutin. The organic acid standards included fumaric acid, malic acid, oxalic acid, and succinic acid. The solvents used for chromatographic analysis, including acetonitrile, methanol, and acetic acid, were of HPLC grade.

### 2.2. Colchicine Treatment

Cladodes of *O. ficus-indica* were subjected to colchicine treatments according to a 3 × 3 factorial arrangement in a randomized complete block design (RCBD) with three blocks. Factor A comprised three maturity stages—1, 3, and 6 months after planting (MAP)—whereas Factor B comprised three colchicine concentrations—0% (*w*/*v*, control), 0.5% (*w*/*v*), and 1.0% (*w*/*v*)—with a 24-h exposure period. Plant cultivation and management procedures were performed according to Boonnak et al. (2026) [14]. The experiment was conducted in a greenhouse with three blocks assigned according to physical bench locations to account for minor microclimatic variations, particularly differences in light intensity and temperature (32–35 °C). Each block contained 10 plants per treatment × maturity-stage combination, resulting in 90 plants per block and 270 plants in total. At each maturity stage, 10 plants per treatment combination were harvested from each block. Tissues were manually separated into epidermis and cortex according to Boontiang et al. (2026) [9]. For each treatment × maturity-stage combination within each block, the corresponding freeze-dried epidermis and cortex tissues were separately pooled to obtain one composite biological sample per block, yielding three independent biological replicates (*n* = 3). The pooled samples were ground using a 40-mesh sieve and stored at −20 °C until analysis. Each biological composite sample was extracted and analyzed in technical triplicate. The mean of the technical triplicates was used as the experimental unit for statistical analysis.

### 2.3. Cladode Extraction for Bioactive Compounds and Antioxidant Activities

Bioactive compounds and antioxidant activities were extracted following a modified procedure based on Boonarsa et al. (2024) [17]. Briefly, 5 g of powdered sample was mixed with 25 mL of 80% (*v*/*v*) ethanol and shaken continuously at room temperature for 12 h. The resulting mixture was filtered through Whatman No. 1 filter paper, and the filtrate was collected in amber bottles and stored under refrigeration until analysis.

### 2.4. Determination of Total Phenolic Content (TPC)

The total phenolic content was quantified using the Folin–Ciocalteu colorimetric assay according to Carmona-Hernandez et al. (2021) [18] with minor modifications. Briefly, 0.2 mL of extract was mixed with 0.5 mL of 10% Folin–Ciocalteu reagent. The contents were mixed by manual shaking for 15–20 s. After 1 min, 2.25 mL of 7% (*w*/*v*) sodium carbonate solution was added. The reaction mixture was incubated in the dark at room temperature for 1.5 h, and the absorbance was measured at 725 nm using a UV–visible spectrophotometer (DR 2800, Hach, Loveland, CO, USA). The values were calculated using a gallic acid standard curve and expressed as mg gallic acid equivalents (GAE) per 100 g dry weight (DW).

### 2.5. Determination of Total Flavonoid Content (TFC)

The total flavonoid content (TFC) was determined using the aluminum chloride colorimetric method according to Boonarsa et al. (2024) [17], with slight modifications. Briefly, 1.0 mL of the extract was mixed with 0.15 mL of 5% (*w*/*v*) sodium nitrite (NaNO_2_) solution and allowed to stand for 5 min. Subsequently, 0.3 mL of 10% (*w*/*v*) AlCl_3_·6H_2_O solution was added, and the mixture was incubated for 6 min. Afterward, 1.0 mL of 1 M NaOH was added, and the mixture was thoroughly mixed. The absorbance was measured at 510 nm using a UV–visible spectrophotometer (DR 2800, Hach, Loveland, CO, USA). The total flavonoid content was calculated using a quercetin standard curve and expressed as mg quercetin equivalents (QE) per 100 g DW.

### 2.6. Determination of DPPH Radical-Scavenging Activity

The antioxidant activity was determined using the 2,2–diphenyl–1–picrylhydrazyl (DPPH) radical-scavenging assay according to Boontiang et al. (2026) [9], with slight modifications. Briefly, 0.5 mL of the extract was mixed with 4.5 mL of 0.06 mM DPPH solution. The reaction mixture was incubated in the dark at room temperature for 30 min, and the absorbance was measured at 517 nm using a UV–visible spectrophotometer (DR 2800, Hach, Loveland, CO, USA). Antioxidant activity was quantified using an L-ascorbic acid standard curve and expressed as mg L-ascorbic acid equivalents per 100 g DW.

### 2.7. Determination of Ferric Reducing Antioxidant Power (FRAP)

The ferric reducing antioxidant power (FRAP) assay was performed according to Yimer et al. (2023) [19], with minor modifications. Briefly, 0.1 mL of the extract was mixed with 4.5 mL of freshly prepared FRAP reagent. The reaction mixture was incubated at room temperature for 10 min, and the absorbance was measured at 593 nm using a UV–visible spectrophotometer (DR 2800, Hach, Loveland, CO, USA). Antioxidant activity was calculated using a ferrous sulfate (FeSO_4_) standard curve and expressed as mg FeSO_4_ equivalents per 100 g DW.

### 2.8. Determination of Phenolic Acids and Flavonoids by HPLC

Phenolic acids and flavonoids were analyzed using an HPLC system (Series 20, Shimadzu, Kyoto, Japan) following a modified method adapted from Chumroenphat et al. (2025) [20]. Chromatographic separation was performed at 38 °C with an injection volume of 20 µL. UV–visible detection was carried out at 280 nm for hydroxybenzoic acids, 320 nm for hydroxycinnamic acids, and 370 nm for flavonoids. Compound identification was based on comparison of the retention times and UV spectra with those of authenticated reference standards.

### 2.9. Determination of Organic Acids by HPLC

Organic acids were analyzed by HPLC following a modified sample preparation procedure adapted from Chumroenphat et al. (2025) [20]. Separation was performed under isocratic conditions using 0.05 M sulfuric acid as the mobile phase at a flow rate of 0.5 mL/min on an Aminex^®^ HPX-87H column (300 × 7.8 mm, 5.0 µm, Bio-Rad Laboratories, Inc., Hercules, CA, USA) maintained at 80 °C. The injection volume was 20 µL, and detection was carried out using a refractive index detector. Individual organic acids were identified by comparing their retention times with those of authentic standards, and the results were expressed as mg/g DW.

### 2.10. Fourier Transform Infrared Spectroscopy (FTIR) Analysis

The functional groups of the samples were analyzed using an ATR–FTIR spectrometer (INVENIO S/LUMOS II, Bruker, Fällanden, Switzerland). Approximately 1 mg of freeze-dried sample powder was placed directly onto the ATR crystal without KBr pellet preparation. Spectra were recorded over the wavenumber range of 4000–400 cm^−1^ at a resolution of 4 cm^−1^. Baseline correction was applied to all spectra prior to analysis according to Bouaouine et al. (2018) [21]. The resulting spectra were used to identify functional groups and compare the structural characteristics of the samples among different treatments.

### 2.11. X-Ray Diffraction (XRD) Analysis

The crystalline characteristics of the samples were analyzed using a D8 Advance X-ray diffractometer (Bruker, Karlsruhe, Germany). The instrument was operated at 40 kV and 40 mA, and diffraction patterns were recorded over a 2θ range of 5–40° at a scanning rate of 2° min^−1^, following the method described by Ouhammou et al. (2024) [22].

### 2.12. Statistical Analysis

All results are expressed as mean ± standard deviation (SD) of three biological replicates (*n* = 3). Prior to statistical analysis, data were tested for normality and homogeneity of variance using the Shapiro–Wilk and Levene’s tests, respectively. A two-way analysis of variance (ANOVA) was performed to evaluate the effects of colchicine concentration, maturity stage, and their interaction on all measured parameters. When significant differences were detected, means were compared using Duncan’s multiple range test at *p* < 0.05. Statistical analyses were conducted separately for the epidermis and cortex; therefore, direct statistical comparisons between tissue types were not performed. Pearson’s correlation analysis and heatmap visualization were performed using MetaboAnalyst 6.0 [23] to investigate the relationships among bioactive compounds, antioxidant activities, phenolic acids, flavonoids, and organic acids. Graphs were prepared using Origin 2022 (OriginLab Corporation, Northampton, MA, USA), and statistical analyses were performed using IBM SPSS Statistics version 17 (IBM Corp., Armonk, NY, USA).

## 3. Results and Discussion

### 3.1. Bioactive Compounds and Antioxidant Activities

Colchicine treatment significantly affected bioactive compounds and antioxidant activities, with responses depending on colchicine concentration and maturity stage (Table 1 and Table 2). The epidermis generally showed higher TPC, TFC, and antioxidant activities than the cortex across the treatments and maturity stages. TPC ranged from 942.67 to 1292.72 mg GAE/100 g DW in the epidermis and 1092.10 to 1286.55 mg GAE/100 g DW in the cortex, whereas TFC varied from 509.42 to 1322.55 mg QE/100 g DW and 153.36 to 717.75 mg QE/100 g DW, respectively. At the early maturity stage (1 month), 0.5% colchicine increased TPC by approximately 6.5% in both tissues compared with the untreated control (0%), whereas 1.0% colchicine reduced TPC by approximately 5–6% in the epidermis. As the cladodes progressed through the different maturity stages, TPC decreased at the intermediate maturity stage (3 months) before partially recovering at the late maturity stage (6 months). In contrast, TFC exhibited an opposite trend, reaching its highest value under 0.5% colchicine at the late maturity stage, representing an increase of approximately 45% compared with the corresponding control. These findings indicate that phenolic and flavonoid accumulation varied with maturity stage and showed concentration-dependent responses to colchicine treatment.

**Figure 1:**
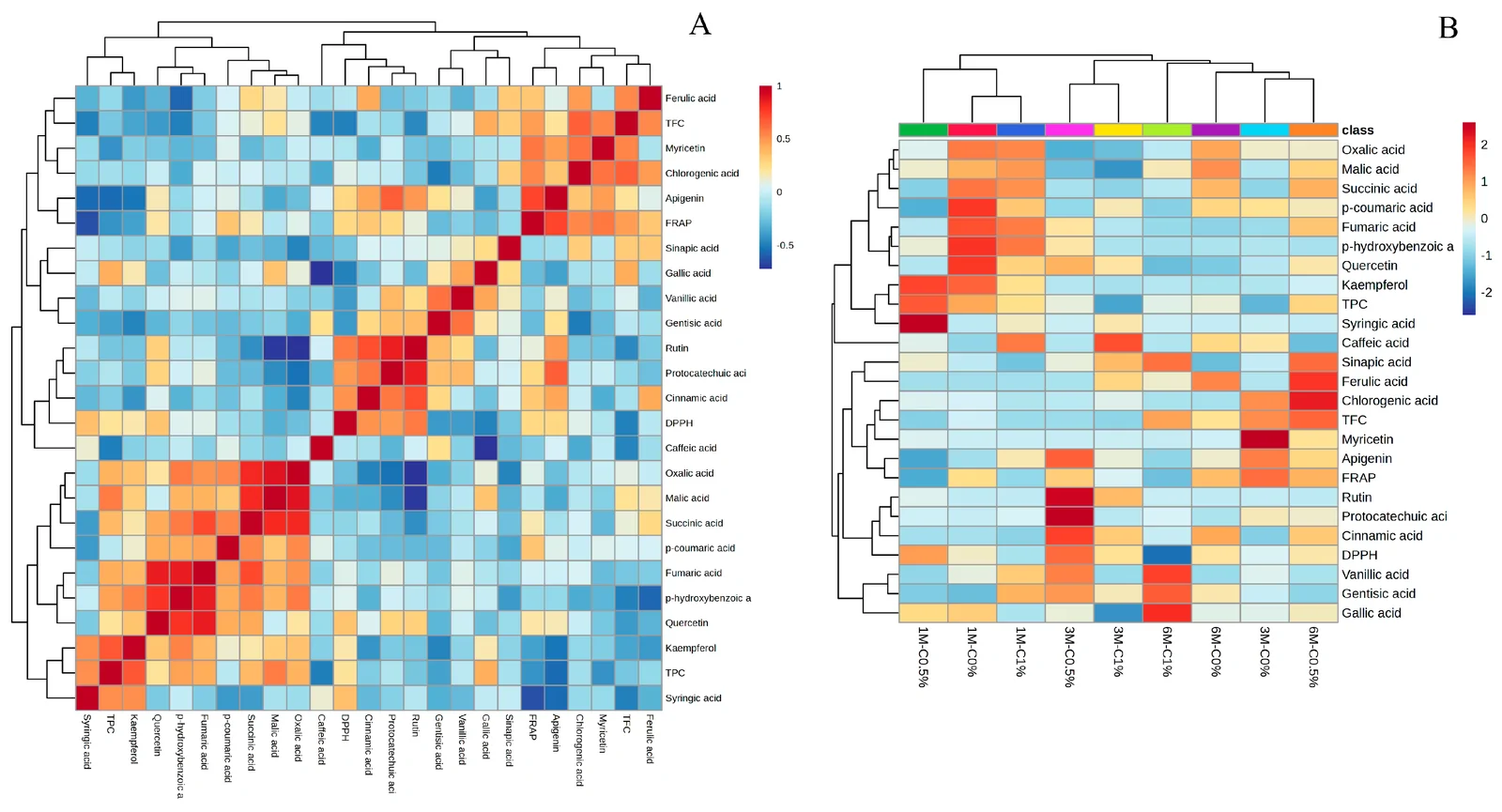
Pearson’s correlation matrix (**A**) and heatmap (**B**) illustrating the relationships and distribution patterns of phenolic acids, flavonoids, organic acids, total phenolic content, total flavonoid content, and antioxidant activities in the epidermis of *O. ficus-indica* cladodes following colchicine treatment during different maturity stages.

**Figure 2:**
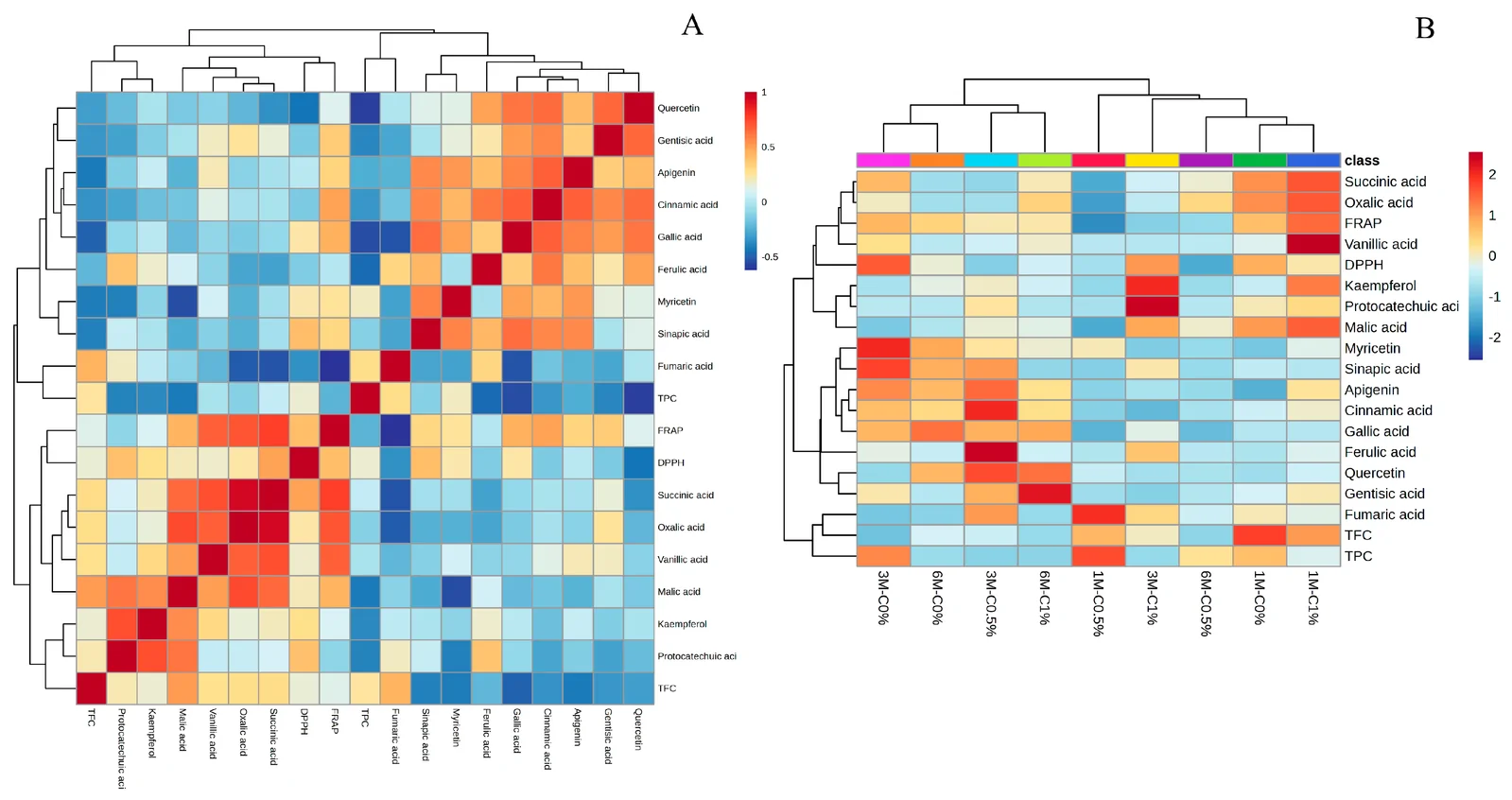
Pearson’s correlation matrix (**A**) and heatmap (**B**) illustrating the relationships and distribution patterns of phenolic acids, flavonoids, organic acids, total phenolic content, total flavonoid content, and antioxidant activities in the cortex of *O. ficus-indica* cladodes following colchicine treatment at different maturity stages.

Antioxidant activities also varied among treatments. DPPH radical-scavenging activity showed a relatively more pronounced response to 0.5% colchicine, whereas FRAP activity did not follow the same pattern and reached its maximum in the untreated epidermis at 3 months. This suggests that the antioxidant response to colchicine was not strictly dose-proportional. The contrasting responses between 0.5% and 1.0% colchicine may reflect concentration-dependent physiological responses, potentially involving differences in secondary metabolite accumulation and cellular metabolic processes. However, these mechanisms were not directly investigated in the present study and therefore require further study. The generally higher accumulation of bioactive compounds in the epidermis than in the cortex is consistent with the protective role of the epidermis against ultraviolet radiation, dehydration, and oxidative stress [24,25]. In contrast, the cortex contained comparatively higher levels of organic acids, particularly malic acid, consistent with its role as a storage tissue in succulent plants [26,27]. These tissue-specific patterns were further supported by HPLC analysis (Table 3 and Table 4) and Pearson’s correlation analysis (Figure 1 and Figure 2).

### 3.2. Phenolic Acid Composition

The phenolic acid profile of *O. ficus-indica* cladodes was significantly influenced by colchicine concentration, maturity stage, and tissue type (Table 3 and Table 4). The epidermis contained higher total phenolic acids (882–2047 mg/100 g DW) than the cortex (166.23–1773.97 mg/100 g DW). Gallic acid and gentisic acid were the predominant phenolic acids in both tissues, whereas chlorogenic acid was detected exclusively in the epidermis. In the epidermis, total phenolic acids increased approximately 1.7-fold, reaching the highest level under 1.0% colchicine at the late maturity stage (6 months). This increase was mainly associated with higher gallic and gentisic acid contents, which increased by approximately 64% and 24%, respectively, compared with the untreated control at the same maturity stage. Ferulic acid and chlorogenic acid also responded to colchicine treatment, reaching their highest concentrations under 0.5% colchicine at 6 months (92.56 and 129.10 mg/100 g DW, respectively). A similar pattern was observed in the cortex, where total phenolic acids reached 1773.97 mg/100 g DW under 1.0% colchicine at 6 months, representing an approximately 79% increase compared with the corresponding control. These results indicate that individual phenolic acids responded differently to colchicine concentration rather than showing a uniform accumulation pattern. The tissue-specific pattern was further supported by the absence of caffeic, chlorogenic, *p*-coumaric, *p*-hydroxybenzoic, and syringic acids in the cortex.

The predominance of gallic and gentisic acids is consistent with previous reports on *O. ficus-indica* cladodes [9]. The exclusive detection of chlorogenic acid and higher ferulic acid accumulation in the epidermis may be associated with the protective role of this tissue against environmental stress [28]. Similar increases in phenolic acids following colchicine treatment have also been reported in *Cyclocarya paliurus* [15], *Foeniculum vulgare* Mill. [29], and *Salvia officinalis* [30], although the magnitude and composition of the accumulated phenolics differed among species. The higher accumulation of total phenolic acids at 1.0% colchicine, particularly at 6 months, together with the responses of individual phenolic acids to 0.5% colchicine, further supports a concentration-dependent but non-uniform response. Phenolic acids are synthesized through the shikimate and phenylpropanoid pathways, and environmental or chemical stimuli may influence their accumulation [31,32]. However, these pathways were not directly investigated in the present study; therefore, the observed changes should be interpreted as associations rather than confirmed metabolic mechanisms.

### 3.3. Flavonoid Composition

The flavonoid profile was markedly influenced by colchicine concentration and maturity stage (Table 3 and Table 4). The epidermis generally contained substantially higher flavonoid contents than the cortex, consistent with the higher TFC observed in the colorimetric assay. Rutin and apigenin were the predominant flavonoids in the epidermis, whereas apigenin and kaempferol were the major compounds in the cortex. Rutin was detected exclusively in the epidermis, indicating a distinct tissue-specific distribution. In the epidermis, total flavonoid content determined by HPLC ranged from 339.30 to 1256.88 mg/100 g DW, representing an approximately 3.7-fold difference between the lowest and highest values. The highest numerical value was observed at the intermediate maturity stage (3 months) in the untreated control (1256.88 mg/100 g DW), which was not significantly different from the 0.5% colchicine treatment (1249.73 mg/100 g DW). This increase was mainly associated with apigenin and rutin, while myricetin also increased at this stage. However, individual flavonoids showed different responses to colchicine and maturity. Quercetin decreased with increasing maturity, whereas rutin and apigenin showed higher accumulation at 3 months. At 6 months, most flavonoids tended to decline. In the cortex, total flavonoid content ranged from 35.16 to 93.59 mg/100 g DW, approximately 10-fold lower than that in the epidermis, with the highest value observed under 0.5% colchicine at 3 months. These results indicate that flavonoid accumulation varied according to tissue type and maturity stage, with concentration-dependent responses to colchicine observed for some individual flavonoids.

The predominance of rutin and apigenin in the epidermis is consistent with previous reports on *O. ficus-indica*, in which flavonoids preferentially accumulate in outer tissues because of their protective roles against environmental stress [7,33]. The exclusive detection of rutin in the epidermis further supports this tissue-specific distribution. Increased flavonoid accumulation following colchicine treatment has also been reported in *Foeniculum vulgare* Mill. [29], *Gloriosa superba* L. [34], and *Catharanthus roseus* (Linn.) G. Don [35], although responses varied with species, colchicine concentration, and developmental stage. The high HPLC-derived total flavonoid contents observed at 3 months in both the untreated control and 0.5% colchicine treatment, together with the different responses of individual flavonoids, further support a concentration-dependent but non-uniform response to colchicine. Flavonoids are synthesized through the phenylpropanoid pathway and are associated with plant antioxidant defense [36]. The observed increases in several flavonoids were consistent with changes in TFC and antioxidant activities; however, these associations do not establish a direct causal relationship. The specific metabolic mechanisms underlying the concentration- and maturity-dependent responses remain to be investigated.

### 3.4. Organic Acid Composition

Organic acid contents were significantly affected by colchicine concentration and maturity stage (Table 5 and Table 6). The cortex generally contained higher concentrations of organic acids than the epidermis, with malic acid being the predominant organic acid in both tissues (3.92–110.88 mg/g DW). This finding is consistent with previous studies on *O. ficus-indica*, in which malic acid was identified as the principal organic acid in cladodes [26]. At the early maturity stage (1 month), 0.5% colchicine reduced most organic acids by approximately 40–60% compared with the untreated control, whereas 1.0% colchicine maintained or slightly increased malic acid content, particularly in the cortex, where it was approximately 12% higher than the control. These results indicate that individual organic acids responded differently to colchicine concentration, with distinct patterns observed between tissues and maturity stages. Similar concentration-dependent changes in primary metabolites following colchicine treatment have been reported in *Cyclocarya paliurus* [15] and *Stevia rebaudiana* [16].

As the cladodes progressed through maturity, most organic acids declined markedly at the intermediate stage (3 months), particularly fumaric and succinic acids, before partially recovering at the late stage (6 months). Similar developmental changes in organic acid composition have been reported in *O. ficus-indica* cladodes and other fruits [37,38]. The relatively high accumulation of organic acids during early development may be associated with the metabolic requirements of actively growing tissues [39]. The consistently higher organic acid contents in the cortex further indicate a tissue-specific pattern of metabolite distribution within the cladodes. Although colchicine influenced individual organic acids, malic acid remained predominant across treatments and maturity stages, suggesting that colchicine altered the abundance of specific organic acids without substantially changing the overall organic acid profile.

### 3.5. Correlation and Heatmap Analysis of Bioactive Compounds and Metabolites

Pearson’s correlation analysis (Figure 1A and Figure 2A) revealed distinct relationships among bioactive compounds, antioxidant activities, phenolic acids, flavonoids, and organic acids in both the epidermis and cortex of *O. ficus-indica* cladodes. In both tissues, TPC and TFC were positively correlated with DPPH and FRAP, indicating that antioxidant activities were associated with the accumulation of phenolic compounds and flavonoids. Several phenolic acids, particularly gallic acid, gentisic acid, chlorogenic acid, and ferulic acid, as well as flavonoids such as rutin, apigenin, and quercetin, also showed positive correlations with antioxidant activities. These results were consistent with the HPLC and spectrophotometric analyses and suggest that antioxidant activity was associated with the combined accumulation of multiple phenolic compounds rather than a single constituent. Similar relationships have been reported in *Opuntia* species and other plant materials [7,32]. In contrast, most organic acids, particularly malic, succinic, fumaric, and oxalic acids, showed weak or negative correlations with phenolic compounds and antioxidant activities and formed a distinct cluster from the secondary metabolites. This pattern is consistent with the different metabolic roles of organic acids and phenolic compounds during plant development and tissue maturation [40].

**Figure 3:**
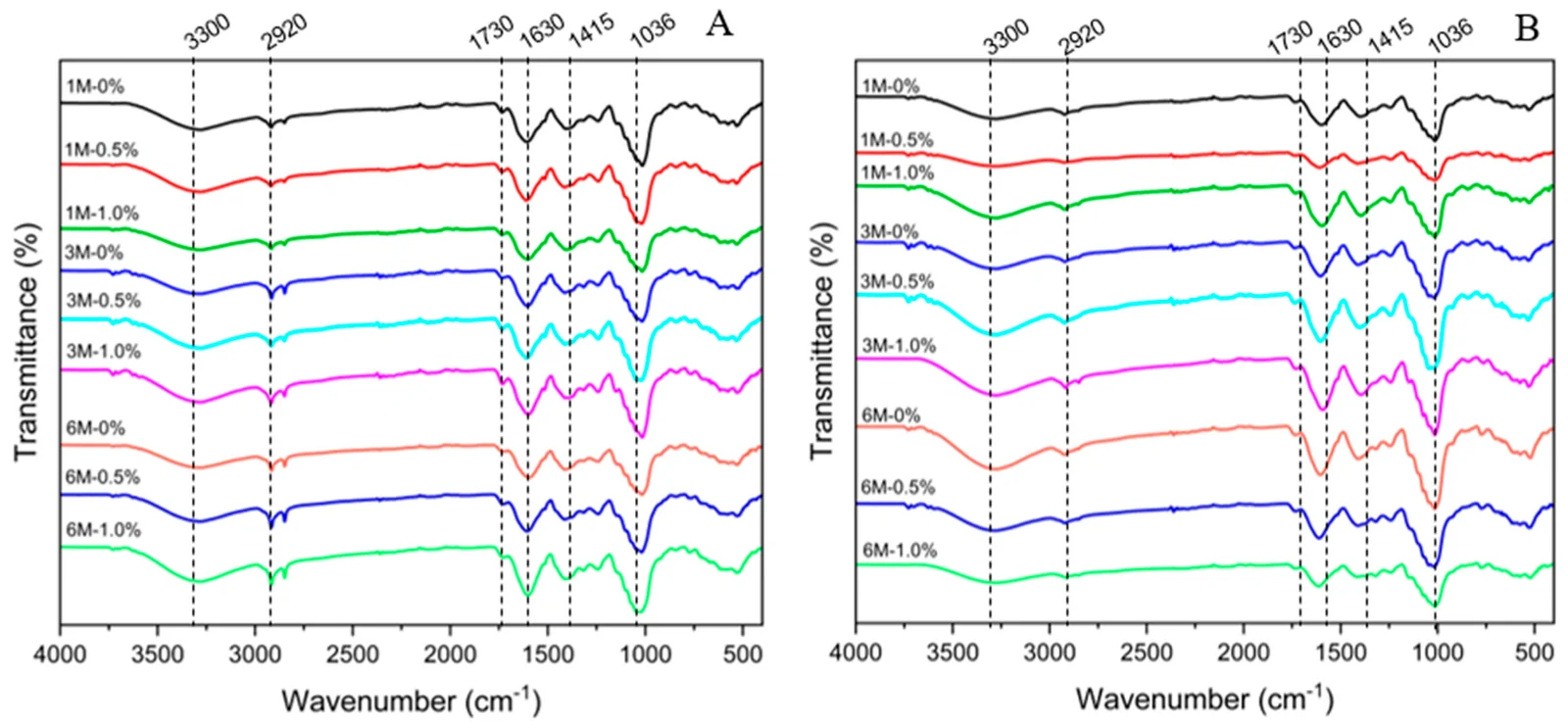
FTIR spectra of the epidermis (**A**) and cortex (**B**) of *O. ficus-indica* cladodes following treatment with 0, 0.5, and 1.0% colchicine at different maturity stages (1, 3, and 6 months).

**Figure 4:**
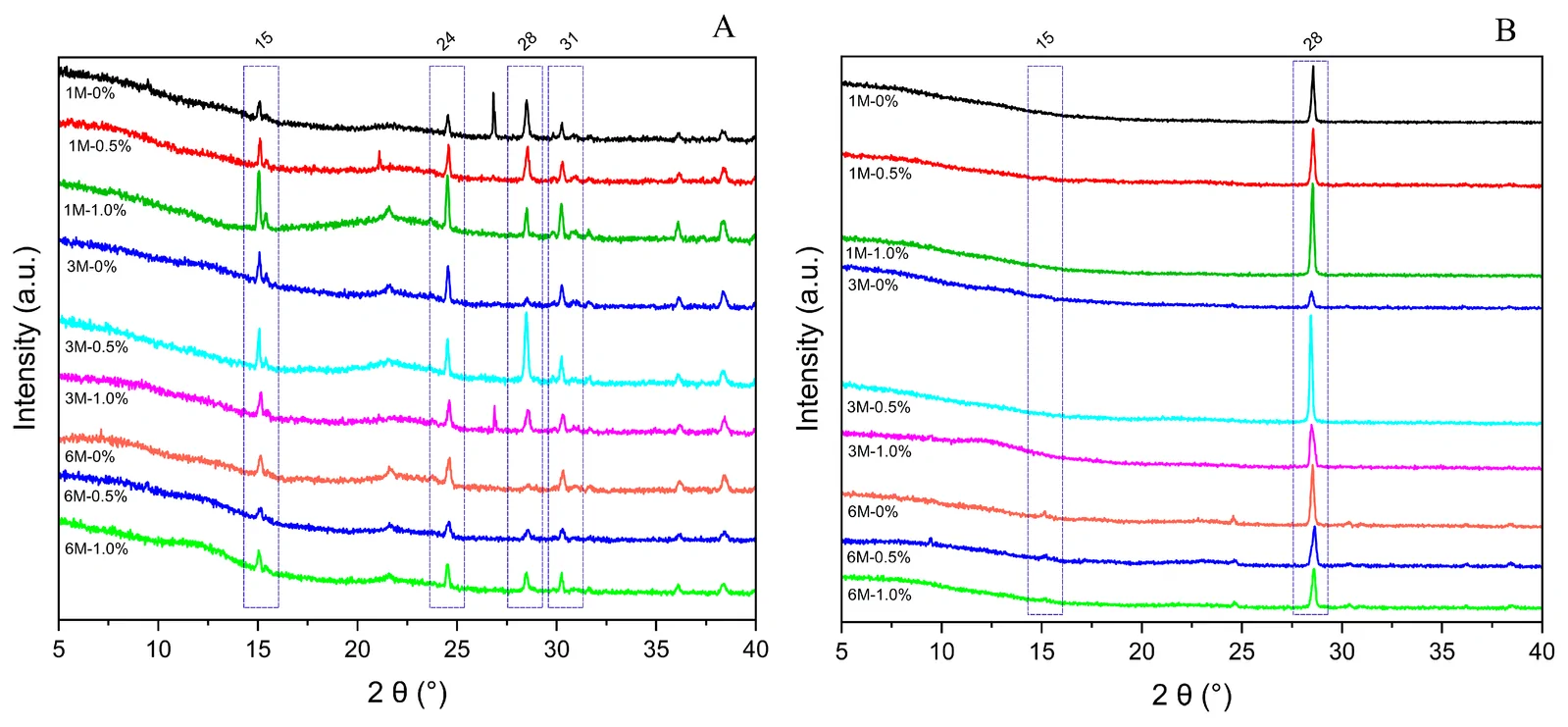
X-ray diffraction patterns of the epidermis (**A**) and cortex (**B**) of *O. ficus-indica* cladodes following treatment with 0, 0.5, and 1.0% colchicine at different maturity stages (1, 3, and 6 months).

The heatmap analysis (Figure 1B and Figure 2B) further revealed distinct metabolite accumulation patterns among treatments. Samples at the late maturity stage (6 months), particularly those treated with 0.5% and 1.0% colchicine, were generally associated with higher levels of phenolic compounds, flavonoids, TPC, TFC, and antioxidant activities, whereas samples at the early maturity stage (1 month) showed relatively higher concentrations of several organic acids. The clustering pattern further indicated that maturity stage was associated with substantial differences in metabolite accumulation. These analyses support the combined association of maturity stage, colchicine concentration, and tissue-specific patterns with metabolite accumulation in *O. ficus-indica* cladodes. When considered together with the FTIR and XRD results (Figure 3 and Figure 4), the observed metabolite differences occurred alongside only minor variations in spectral and diffraction patterns, with no substantial changes in the major functional groups or crystalline characteristics. This suggests that metabolite accumulation varied within a relatively stable chemical and structural framework of the cladode tissues.

### 3.6. Fourier Transform Infrared Spectroscopy (FTIR)

The FTIR spectra of the epidermis and cortex of *O. ficus-indica* cladodes at different maturity stages are presented in Figure 3. All samples exhibited broadly similar absorption patterns, indicating that colchicine treatment did not introduce detectable new functional groups. However, slight variations in band intensity were observed among tissues, colchicine concentrations, and maturity stages, which may reflect differences in the relative contribution of existing chemical constituents. Comparable FTIR profiles have been reported for *Opuntia* cladodes and have been associated with polysaccharides, phenolic compounds, organic acids, and structural carbohydrates [26,39]. A broad absorption band at approximately 3300–3400 cm^−1^, corresponding to O–H stretching vibrations, was observed in all samples and is associated with hydroxyl-containing compounds, including phenolic compounds, flavonoids, cellulose, and pectic polysaccharides [41,42]. Bands at approximately 2850–2920 cm^−1^ were assigned to C–H stretching vibrations of aliphatic groups, while bands near 1730 and 1600–1630 cm^−1^ corresponded mainly to C=O and C=C stretching vibrations, respectively [41,42,43,44]. A strong absorption region between 1150 and 1000 cm^−1^ was also observed, corresponding mainly to C–O and C–O–C stretching vibrations of polysaccharides such as cellulose, hemicellulose, and pectic substances [42,43,44,45]. The similarity of these characteristic bands among treatments indicates that colchicine did not substantially alter the major functional groups of the cladode tissues. Nevertheless, the observed differences in band intensity were observed alongside variations in phenolic acids, flavonoids, and organic acids detected by HPLC, particularly between the epidermis and cortex. Collectively, the FTIR results indicate that the differences in phenolic acids, flavonoids, and organic acids observed among tissues and treatments were not accompanied by substantial changes in the major functional groups. This suggests that the observed metabolite variation occurred within an overall similar chemical framework rather than being accompanied by the formation of new major functional groups.

### 3.7. X-Ray Diffraction (XRD)

The XRD patterns of the epidermis and cortex of *O. ficus-indica* cladodes are presented in Figure 4. Both tissues exhibited similar diffraction patterns across different maturity stages, indicating that colchicine treatment did not substantially alter the crystalline characteristics of the cladode tissues. Minor differences were observed in the relative intensity of several diffraction peaks. The epidermis exhibited diffraction peaks at approximately 2θ = 15°, 22–25°, 28–30°, and 31–32°, whereas the cortex was mainly characterized by peaks near 2θ = 15° and 28–29°. These diffraction features are consistent with plant cell-wall components, including crystalline cellulose and less-ordered polysaccharide regions, and are comparable with previous reports on *Opuntia* cladodes and dietary fibers [46].

Differences among maturity stages were observed mainly in the intensity of peaks associated with cellulose-rich crystalline regions (2θ ≈ 22–23°) and less-ordered polysaccharide regions (2θ ≈ 15–16°). These variations may reflect changes in the relative proportions or organization of crystalline and less-ordered cell-wall components during cladode development rather than changes in the fundamental crystalline structure [46,47]. The diffraction peak near 2θ ≈ 28° may also be associated with mineral constituents naturally present in cactus tissues, including calcium oxalate crystals [39]. Despite these variations, the overall diffraction patterns remained comparable among treatments, indicating that colchicine treatment did not substantially alter the crystalline characteristics of the cladode tissues. These findings suggest that the changes in metabolite accumulation occurred within a relatively stable cell-wall structural framework, although subtle changes in the organization of crystalline and less-ordered components cannot be excluded. Collectively, the FTIR and XRD results indicate that the observed biochemical differences occurred without substantial changes in the major functional groups or crystalline characteristics of the cladode tissues.

## 4. Conclusions

This study showed that colchicine treatment and maturity stage were associated with differences in metabolite accumulation in *O. ficus-indica* cladodes. The epidermis generally contained higher concentrations of phenolic compounds, flavonoids, and antioxidant activities than the cortex, whereas the cortex accumulated higher levels of organic acids, with malic acid remaining the predominant organic acid. The late maturity stage (6 months) was generally associated with higher levels of phenolic compounds, flavonoids, and antioxidant activities, while the early maturity stage (1 month) was characterized by higher organic acid contents. Pearson’s correlation analysis revealed positive associations between phenolic compounds, flavonoids, and antioxidant activities, whereas FTIR and XRD analyses indicated that the observed biochemical differences occurred without substantial changes in the major functional groups or crystalline characteristics of the cladode tissues. Among the treatments, 0.5% colchicine was generally associated with higher flavonoid contents and antioxidant activities, whereas 1.0% colchicine was associated with increased levels of several phenolic acids. Although polyploid and cytochimeric plants were confirmed in our previous study, ploidy levels were not re-evaluated in the present study; therefore, the observed metabolite differences cannot be conclusively attributed to genome dosage effects. These findings indicate that metabolite accumulation and functional quality varied according to maturity stage, tissue, and colchicine concentration in *O. ficus-indica* cladodes and provide a basis for further investigation of their potential food and nutraceutical applications.

From an application perspective, the epidermis represents a promising source of phenolic compounds and antioxidant constituents for functional food and plant-based ingredient applications, whereas the cortex, with its higher organic acid content, may provide useful characteristics for food ingredient development. Future studies should further investigate the relationship between ploidy level and metabolite accumulation, together with the underlying metabolic and molecular mechanisms, bioavailability, and biological activities of the identified phytochemicals. Such studies would help clarify the contribution of polyploidy to the biochemical characteristics of *O. ficus-indica* and further support its development as a high value functional food ingredient.

## Figures and Tables

**Table 1:** Effects of colchicine treatment and different maturity stages on bioactive compounds and antioxidant activities in the epidermis of *O. ficus-indica* cladodes.

Maturity Stages	Colchicine Concentration	TPC (mg GAE/100 g DW)	TFC (mg QE/100 g DW)	DPPH (mg L-Ascorbic Acid/100 g DW)	FRAP (mg FeSO_4_/100 g DW)
1 Month	0%	1213.76 ± 24.61 ^b^	716.49 ± 3.67 ^e^	153.77 ± 2.31 ^cd^	1406.78 ± 3.74 ^e^
	0.5%	1292.72 ± 11.86 ^a^	509.42 ± 4.72 ^h^	161.57 ± 2.31 ^b^	1015.44 ± 7.35 ^i^
	1.0%	1144.31 ± 5.28 ^d^	561.19 ± 5.79 ^f^	148.62 ± 2.62 ^e^	1143.71 ± 5.10 ^g^
3 Months	0%	965.82 ± 9.77 ^f^	1212.70 ± 25.22 ^b^	151.67 ± 2.42 ^d^	1651.06 ± 11.58 ^a^
	0.5%	1096.22 ± 3.38 ^e^	568.76 ± 9.53 ^f^	164.96 ± 1.81 ^a^	1452.53 ± 10.20 ^d^
	1.0%	942.67 ± 3.04 ^g^	527.1 ± 3.14 ^g^	155.60 ± 3.89 ^c^	1275.25 ± 2.00 ^f^
6 Months	0%	1099.05 ± 5.18 ^e^	910.93 ± 9.53 ^d^	155.27 ± 4.31 ^c^	1490.11 ± 9.28 ^c^
	0.5%	1157.69 ± 3.94 ^c^	1322.55 ± 9.53 ^a^	154.86 ± 4.62 ^c^	1517.89 ± 10.68 ^b^
	1.0%	1090.04 ± 6.04 ^e^	1152.1 ± 30.62 ^c^	138.05 ± 3.42 ^f^	1074.26 ± 6.48 ^h^

Values are expressed as mean ± SD (*n* = 3). Means with different letters (^a,b,c,…^) are significantly different at *p* < 0.05 within the same column in the parameter. TPC: total phenolics content; TFC: total flavonoids content; GAE: gallic acid equivalent; QE: quercetin equivalent; DPPH: 2,2-diphenyl-1-picrylhydrazyl; FRAP: ferric reducing antioxidant power; DW: dry weight.

**Table 2:** Effects of colchicine treatment and different maturity stages on bioactive compounds and antioxidant activities in the cortex of *O. ficus-indica* cladodes.

Maturity Stages	Colchicine Concentration	TPC (mg GAE/100 g DW)	TFC (mg QE/100 g DW)	DPPH (mg L-Ascorbic Acid/100 g DW)	FRAP (mg FeSO_4_/100 g DW)
1 Month	0%	1210.42 ± 27.53 ^b^	717.75 ± 5.79 ^a^	116.16 ± 2.34 ^b^	803.02 ± 8.72 ^b^
	0.5%	1286.55 ± 10.36 ^a^	513.21 ± 9.53 ^c^	99.48 ± 2.20 ^e^	543.22 ± 2.83 ^g^
	1.0%	1143.03 ± 9.52 ^d^	566.24 ± 9.53 ^b^	108.57 ± 3.11 ^c^	886.36 ± 5.10 ^a^
3 Months	0%	1247.71 ± 27.78 ^b^	153.36 ± 7.89 ^i^	124.09 ± 1.94 ^a^	812.01 ± 6.48 ^b^
	0.5%	1092.10 ± 17.12 ^f^	292.25 ± 2.19 ^f^	96.84 ± 2.61 ^e^	739.30 ± 11.05 ^d^
	1.0%	1099.05 ± 2.92 ^f^	373.06 ± 5.79 ^d^	118.26 ± 3.51 ^b^	626.55 ± 3.74 ^f^
6 Months	0%	1102.14 ± 3.12 ^e^	312.45 ± 2.19 ^e^	106.19 ± 3.33 ^cd^	777.70 ± 2.45 ^c^
	0.5%	1178.52 ± 10.08 ^c^	197.55 ± 4.37 ^h^	92.16 ± 2.35 ^g^	642.08 ± 5.10 ^e^
	1.0%	1092.62 ± 1.18 ^f^	213.96 ± 7.89 ^g^	103.48 ± 2.31 ^d^	743.38 ± 2.45 ^d^

Values are expressed as mean ± SD (*n* = 3). Means with different letters (^a,b,c,…^) are significantly different at *p* < 0.05 within the same column in the parameter. TPC: total phenolics content; TFC: total flavonoids content; GAE: gallic acid equivalent; QE: quercetin equivalent; DPPH: 2,2-diphenyl-1-picrylhydrazyl; FRAP: ferric reducing antioxidant power; DW: dry weight.

**Table 3:** Effects of colchicine treatment and different maturity stages on phenolic acid and flavonoid profiles in the epidermis of *O. ficus-indica* cladodes (mg/100 g dry weight).

Maturity Stages		1 Month			3 Months			6 Months	
Colchicine Concentration	0%	0.5%	1.0%	0%	0.5%	1.0%	0%	0.5%	1.0%
Phenolic acid content									
Caffeic acid	1.97 ± 0.10 ^e^	4.06 ± 0.05 ^d^	8.78 ± 1.61 ^b^	5.23 ± 0.43 ^c^	2.86 ± 0.16 ^e^	9.98 ± 0.15 ^a^	5.90 ± 0.07 ^c^	1.02 ± 0.10 ^e^	2.76 ± 0.58 ^de^
Chlorogenic acid	20.66 ± 1.05 ^d^	23.92 ± 0.65 ^c^	6.93 ± 0.07 ^f^	84.01 ± 7.31 ^b^	11.63 ± 0.84 ^e^	25.64 ± 0.69 ^c^	4.30 ± 0.02 ^g^	129.10 ± 5.31 ^a^	5.49 ± 0.29 ^g^
Cinnamic acid	26.85 ± 1.45 ^e^	25.88 ± 0.11 ^e^	19.17 ± 0.62 ^f^	17.38 ± 1.34 ^f^	110.06 ± 10.07 ^a^	67.52 ± 4.53 ^c^	80.48 ± 6.01 ^b^	68.24 ± 2.15 ^c^	35.26 ± 1.80 ^d^
Ferulic acid	0.39 ± 0.01 ^g^	0.47 ± 0.01 ^g^	1.30 ± 0.10 ^g^	5.26 ± 0.14 ^e^	3.27 ± 0.20 ^f^	40.48 ± 0.79 ^c^	67.77 ± 1.15 ^b^	92.56 ± 1.14 ^a^	24.80 ± 0.39 ^d^
Gallic acid	424.71 ± 1.40 ^b^	396.12 ± 3.86 ^c^	213.77 ± 18.33 ^f^	295.5 ± 8.51 ^e^	314.58 ± 27.33 ^e^	23.41 ± 0.71 ^g^	297.05 ± 17.08 ^e^	337.46 ± 6.13 ^d^	695.90 ± 1.38 ^a^
Gentisic acid	351.28 ± 28.20 ^h^	407.55 ± 21.21 ^g^	983.22 ± 39.55 ^c^	597.58 ± 56.65 ^e^	1059.97 ± 59.38 ^b^	749.64 ± 15.31 ^d^	764.66 ± 38.68 ^d^	448.63 ± 13.04 ^f^	1220.12 ± 52.09 ^a^
*p*-coumaric acid	2.16 ± 0.11 ^a^	0.05 ± 0.00 ^g^	1.31 ± 0.09 ^b^	1.10 ± 0.11 ^c^	0.39 ± 0.01 ^e^	0.98 ± 0.02 ^d^	1.21 ± 0.01 ^c^	0.97 ± 0.02 ^d^	0.33 ± 0.01 ^f^
*p*-hydroxybenzoic acid	35.27 ± 0.27 ^a^	11.56 ± 0.27 ^d^	28.39 ± 0.88 ^b^	5.42 ± 0.52 ^e^	14.51 ± 0.44 ^c^	5.17 ± 0.02 ^e^	5.06 ± 0.26 ^e^	4.60 ± 0.34 ^ef^	4.30 ± 0.41 ^f^
Protocatechuic acid	3.59 ± 0.17 ^f^	4.75 ± 0.08 ^e^	6.02 ± 0.01 ^d^	10.88 ± 1.08 ^b^	47.28 ± 2.57 ^a^	2.24 ± 0.10 ^g^	0.74 ± 0.03 ^h^	9.79 ± 0.10 ^c^	5.98 ± 0.05 ^d^
Sinapic acid	1.52 ± 0.01 ^f^	2.32 ± 0.16 ^d^	0.43 ± 0.01 ^g^	1.60 ± 0.11 ^f^	2.19 ± 0.02 ^e^	3.63 ± 0.11 ^c^	0.30 ± 0.01 ^h^	4.87 ± 0.01 ^a^	4.82 ± 0.02 ^b^
Syringic acid	2.05 ± 0.15 ^d^	76.55 ± 3.15 ^a^	14.43 ± 0.47 ^c^	4.27 ± 0.32 ^d^	4.19 ± 0.09 ^d^	16.70 ± 0.71 ^d^	3.03 ± 0.04 ^d^	2.85 ± 0.45 ^d^	4.15 ± 0.14 ^d^
Vanillic acid	12.51 ± 0.18 ^d^	0.83 ± 0.04 ^f^	23.27 ± 1.27 ^c^	11.29 ± 0.65 ^d^	34.73 ± 0.43 ^b^	1.83 ± 0.01 ^f^	1.90 ± 0.04 ^f^	4.08 ± 0.06 ^e^	43.82 ± 3.04 ^a^
Total	882.95 ± 26.66 ^g^	954.06 ± 23.56 ^f^	1307.04 ± 55.01 ^c^	1039.53 ± 71.33 ^e^	1605.66 ± 54.99 ^b^	947.22 ± 57.73 ^d^	1232.39 ± 49.71 ^d^	1104.17 ± 16.51 ^e^	2047.73 ± 46.16 ^a^
Flavonoid content									
Apigenin	447.33 ± 27.58 ^d^	241.61 ± 9.83 ^f^	682.62 ± 29.04 ^c^	1009.76 ± 72.69 ^a^	1087.28 ± 73.76 ^a^	626.07 ± 56.27 ^c^	644.82 ± 22.57 ^c^	768.45 ± 11.42 ^b^	400.71 ± 12.98 ^e^
Kaempferol	8.00 ± 0.54 ^b^	8.43 ± 0.02 ^a^	5.91 ± 0.31 ^c^	4.36 ± 0.06 ^e^	4.45 ± 0.04 ^e^	4.26 ± 0.14 ^e^	4.29 ± 0.05 ^e^	4.81 ± 0.15 ^d^	4.43 ± 0.32 ^e^
Myricetin	17.18 ± 0.22 ^e^	26.12 ± 0.33 ^c^	16.71 ± 1.34 ^e^	180.89 ± 1.55 ^a^	15.07 ± 1.11 ^ef^	17.51 ± 1.65 ^e^	19.88 ± 0.18 ^d^	52.42 ± 2.23 ^b^	15.63 ± 0.22 ^f^
Quercetin	143.90 ± 2.61 ^a^	58.86 ± 2.42 ^e^	95.16 ± 0.72 ^c^	60.40 ± 0.69 ^e^	107.27 ± 0.74 ^b^	85.34 ± 3.94 ^d^	44.37 ± 3.36 ^f^	84.05 ± 0.60 ^d^	38.41 ± 0.99 ^g^
Rutin	1.31 ± 0.09 ^e^	4.28 ± 0.78 ^c^	1.14 ± 0.10 ^f^	1.47 ± 0.13 ^e^	35.66 ± 0.56 ^a^	15.43 ± 0.50 ^b^	1.18 ± 0.07 ^f^	1.12 ± 0.02 ^f^	2.08 ± 0.19 ^d^
Total	617.73 ± 31.70 ^e^	339.30 ± 14.63 ^g^	801.54 ± 29.50 ^c^	1256.88 ± 72.38 ^a^	1249.73 ± 73.77 ^a^	748.59 ± 35.32 ^d^	714.53 ± 25.41 ^d^	910.84 ± 8.74 ^b^	461.26 ± 23.42 ^f^

Values are expressed as mean ± SD (*n* = 3). Means with different letters (^a,b,c…^) are significantly different at *p* < 0.05 within the same row in the parameter.

**Table 4:** Effects of colchicine treatment and different maturity stages on phenolic acid and flavonoid profiles in the cortex of *O. ficus-indica* cladodes (mg/100 g dry weight).

Maturity Stages		1 Month			3 Months			6 Months	
Colchicine Concentration	0%	0.5%	1.0%	0%	0.5%	1.0%	0%	0.5%	1.0%
Phenolic acid content									
Caffeic acid	ND	ND	ND	ND	ND	ND	ND	ND	ND
Chlorogenic acid	ND	ND	ND	ND	ND	ND	ND	ND	ND
Cinnamic acid	5.93 ± 0.16 ^f^	3.46 ± 0.17 ^h^	7.43 ± 0.44 ^e^	10.43 ± 1.01 ^b^	16.23 ± 0.96 ^a^	2.25 ± 0.18 ^i^	9.14 ± 0.20 ^c^	4.65 ± 0.26 ^g^	8.77 ± 0.12 ^d^
Ferulic acid	0.11 ± 0.01 ^i^	0.18 ± 0.01 ^h^	1.68 ± 0.12 ^d^	2.04 ± 0.20 ^c^	12.61 ± 0.39 ^a^	5.19 ± 0.18 ^b^	0.92 ± 0.01 ^f^	0.39 ± 0.02 ^g^	1.19 ± 0.04 ^e^
Gallic acid	268.97 ± 15.66 ^f^	64.13 ± 2.90 ^h^	269.07 ± 23.21 ^f^	645.59 ± 9.18 ^d^	658.88 ± 1.24 ^c^	378.83 ± 10.04 ^e^	814.8 ± 6.70 ^a^	91.45 ± 5.47 ^g^	684.46 ± 1.45 ^b^
Gentisic acid	247.18 ± 6.68 ^e^	96.51 ± 2.93 ^g^	372.06 ± 2.31 ^d^	383.94 ± 6.15 ^c^	614.42 ± 9.24 ^b^	35.55 ± 2.45 ^h^	152.76 ± 7.15 ^f^	147.71 ± 9.77 ^f^	1066.14 ± 8.86 ^a^
*p*-coumaric acid	ND	ND	ND	ND	ND	ND	ND	ND	ND
*p*-hydroxybenzoic acid	ND	ND	ND	ND	ND	ND	ND	ND	ND
Protocatechuic acid	32.43 ± 1.04 ^d^	0.31 ± 0.01 ^i^	45.05 ± 2.24 ^b^	7.15 ± 0.41 ^e^	37.64 ± 2.70 ^c^	130.34 ± 4.99 ^a^	5.38 ± 0.11 ^f^	5.04 ± 0.80 ^g^	4.19 ± 0.03 ^h^
Sinapic acid	1.97 ± 0.17 ^e^	0.71 ± 0.07 ^h^	1.72 ± 0.11 ^e^	7.75 ± 0.57 ^a^	5.82 ± 0.06 ^b^	3.53 ± 0.18 ^d^	5.37 ± 0.08 ^c^	1.12 ± 0.11 ^f^	0.95 ± 0.02 ^g^
Syringic acid	ND	ND	ND	ND	ND	ND	ND	ND	ND
Vanillic acid	5.43 ± 0.08 ^d^	0.93 ± 0.02 ^h^	43.75 ± 2.14 ^a^	12.58 ± 0.43 ^b^	3.77 ± 0.31 ^e^	0.61 ± 0.05 ^i^	1.17 ± 0.11 ^g^	1.55 ± 0.14 ^f^	8.28 ± 0.08 ^c^
Total	562.04 ± 6.26 ^f^	166.23 ± 5.17 ^h^	740.75 ± 63.39 ^e^	1069.45 ± 20.53 ^c^	1349.39 ± 4.88 ^b^	556.30 ± 14.79 ^f^	989.54 ± 11.39 ^d^	251.91 ± 8.54 ^g^	1773.97 ± 11.11 ^a^
Flavonoid content									
Apigenin	2.86 ± 0.16 ^h^	6.23 ± 0.19 ^g^	15.66 ± 0.95 ^d^	23.59 ± 0.25 ^b^	25.90 ± 1.80 ^a^	8.58 ± 0.58 ^e^	19.89 ± 0.35 ^c^	7.12 ± 0.14 ^f^	16.24 ± 0.45 ^d^
Kaempferol	12.67 ± 0.78 ^e^	7.97 ± 0.23 ^h^	32.18 ± 2.19 ^b^	10.06 ± 0.97 ^f^	17.57 ± 0.78 ^c^	40.47 ± 2.16 ^a^	15.84 ± 0.73 ^d^	8.74 ± 0.16 ^g^	13.67 ± 0.86 ^e^
Myricetin	10.12 ± 0.60 ^d^	11.75 ± 0.51 ^c^	11.36 ± 0.58 ^c^	14.54 ± 0.52 ^a^	11.92 ± 0.65 ^c^	10.20 ± 0.27 ^d^	12.93 ± 0.40 ^b^	10.47 ± 0.12 ^d^	11.54 ± 0.14 ^c^
Quercetin	9.51 ± 0.70 ^g^	13.13 ± 0.25 ^e^	14.16 ± 1.09 ^d^	8.69 ± 0.21 ^g^	38.20 ± 1.44 ^a^	10.04 ± 0.96 ^fg^	27.06 ± 1.79 ^c^	10.64 ± 0.49 ^f^	34.37 ± 1.39 ^b^
Rutin	ND	ND	ND	ND	ND	ND	ND	ND	ND
Total	35.16 ± 1.67 ^e^	39.08 ± 1.04 ^d^	73.37 ± 5.92 ^b^	56.88 ± 2.75 ^c^	93.59 ± 8.57 ^a^	69.28 ± 4.49 ^b^	75.73 ± 4.36 ^b^	36.97 ± 1.84 ^e^	75.83 ± 6.75 ^b^

Values are expressed as mean ± SD (*n* = 3). Means with different letters (^a,b,c…^) are significantly different at *p* < 0.05 within the same row in the parameter. ND: Not detected.

**Table 5:** Effects of colchicine treatment and different maturity stages on organic acid profiles in the epidermis of *O. ficus-indica* cladodes (mg/g DW).

Maturity Stages	Colchicine Concentration	Fumaric Acid	Malic Acid	Oxalic Acid	Succinic Acid
1 Month	0%	20.46 ± 0.25 ^a^	49.33 ± 1.83 ^b^	22.85 ± 0.36 ^a^	15.34 ± 0.38 ^a^
	0.5%	3.06 ± 0.01 ^e^	33.58 ± 0.03 ^e^	13.45 ± 0.02 ^e^	1.11 ± 0.01 ^g^
	1.0%	17.49 ± 0.26 ^b^	53.77 ± 3.98 ^a^	22.16 ± 0.51 ^b^	13.80 ± 1.18 ^ab^
3 Months	0%	1.35 ± 0.01 ^g^	23.29 ± 0.31 ^f^	14.71 ± 0.11 ^d^	1.53 ± 0.12 ^f^
	0.5%	9.08 ± 0.06 ^d^	13.25 ± 1.15 ^g^	6.48 ± 0.03 ^h^	2.83 ± 0.02 ^e^
	1.0%	2.90 ± 0.01 ^f^	3.92 ± 0.32 ^h^	7.52 ± 0.01 ^g^	1.38 ± 0.18 ^f^
6 Months	0%	0.39 ± 0.01 ^i^	55.10 ± 0.58 ^a^	20.59 ± 0.03 ^c^	11.63 ± 0.43 ^c^
	0.5%	12.10 ± 0.66 ^c^	42.91 ± 2.37 ^c^	14.77 ± 0.34 ^d^	12.18 ± 2.54 ^bc^
	1.0%	0.91 ± 0.01 ^h^	35.57 ± 0.49 ^d^	11.65 ± 0.01 ^f^	3.66 ± 0.04 ^d^

Values are expressed as mean ± SD (*n* = 3). Means with different letters (^a,b,c,…^) are significantly different at *p* < 0.05 within the same column in the parameter.

**Table 6:** Effects of colchicine treatment and different maturity stages on organic acid profiles in the cortex of *O. ficus-indica* cladodes (mg/g DW).

Maturity Stages	Colchicine Concentration	Fumaric Acid	Malic Acid	Oxalic Acid	Succinic Acid
1 Month	0%	10.06 ± 0.01 ^d^	98.82 ± 0.40 ^b^	23.19 ± 0.01 ^b^	19.76 ± 0.01 ^b^
	0.5%	24.33 ± 0.01 ^a^	40.69 ± 0.99 ^h^	8.05 ± 0.03 ^h^	7.29 ± 0.04 ^i^
	1.0%	8.23 ± 0.01 ^e^	110.88 ± 0.92 ^a^	25.93 ± 0.07 ^a^	22.36 ± 0.23 ^a^
3 Months	0%	1.49 ± 0.01 ^i^	49.48 ± 4.75 ^g^	16.94 ± 0.04 ^e^	17.94 ± 0.12 ^c^
	0.5%	17.26 ± 0.03 ^b^	72.48 ± 1.99 ^de^	12.69 ± 0.12 ^g^	10.06 ± 0.22 ^h^
	1.0%	12.44 ± 0.03 ^c^	95.76 ± 0.08 ^c^	14.18 ± 0.04 ^f^	12.70 ± 0.70 ^f^
6 Months	0%	2.46 ± 0.01 ^h^	59.99 ± 0.38 ^f^	12.67 ± 0.06 ^g^	10.69 ± 0.29 ^g^
	0.5%	6.75 ± 0.02 ^f^	73.41 ± 0.90 ^d^	19.08 ± 0.08 ^d^	14.02 ± 0.40 ^e^
	1.0%	3.40 ± 0.01 ^g^	70.03 ± 0.59 ^e^	19.41 ± 0.02 ^c^	14.72 ± 0.25 ^d^

Values are expressed as mean ± SD (*n* = 3). Means with different letters (^a,b,c,…^) are significantly different at *p* < 0.05 within the same column in the parameter.

## Data Availability

The original contributions presented in this study are included in the article. Further inquiries can be directed to the corresponding author.
